# Prognostic value of HER-2/neu expression in epithelial ovarian cancer: a systematic review and meta-analysis

**DOI:** 10.18632/oncotarget.20657

**Published:** 2017-09-06

**Authors:** Kai Wang, Chenan Guan, Junhui Yu, Xiaoxiao Jin, Ling Sun, Lingzhi Zheng, Liang Xia, Yuquan Zhang

**Affiliations:** ^1^ Department of Obstetrics and Gynecology, Taizhou Hospital of Zhejiang Province, Wenzhou Medical University, Linhai, Zhejiang Province 317000, China; ^2^ Department of Neurosurgery, Zhejiang Cancer Hospital, Hangzhou, Zhejiang Province 310022, China; ^3^ Department of Kidney Internal Medicine, Taizhou Hospital of Zhejiang Province, Wenzhou Medical University, Linhai, Zhejiang Province 317000, China; ^4^ Department of Obstetrics and Gynecology, Affiliated Hospital of Nantong University, Nantong, Jiangsu Province 226001, China

**Keywords:** epithelial ovarian cancer, HER-2/neu, prognosis, meta-analysis

## Abstract

This study aimed to conduct a meta-analysis to investigate the association between human epidermal growth factor receptor 2 (HER-2/neu) expression and survival in patients with epithelial ovarian cancer (EOC). HER-2/neu is one of the most frequently studied molecular biological parameters in EOC, but its prognostic impact has not been fully assessed. PubMed and Embase were searched for studies that reported HER-2/neu expression and survival in patients with EOC. The primary outcome was overall survival (OS), and the secondary outcome was progression-free survival (PFS). Hazard ratios (HRs) with 95% confidence interval (CI) were determined using Mantel–Haenszel random-effects model. Publication bias was investigated using funnel plots and Egger’s test. A total of 56 studies (N=7212) were included in the analysis. The results showed that patients possessing HER-2/neu expression had significant disadvantages in OS (HR = 1.41; 95%CI, 1.31 to 1.51; *P* < 0.001) and PFS (HR = 1.38; 95% CI, 1.23–1.56; *P* < 0.001). The trim-and-fill method, Copas model, and subgroup analyses stratified by the study characteristics confirmed the robustness of the results. The present study findings provided further indication that HER-2/neu expression in patients with EOC has an adverse impact on OS and PFS.

## INTRODUCTION

Epithelial ovarian cancer (EOC) is considered as the second most common malignancy in women. It is the most frequently encountered cause of gynecological cancer death, and the fifth leading cause of cancer deaths in developing countries [1]. A total of 21,880 new cases of ovarian cancer were diagnosed in U.S. that resulted in 13,850 deaths during 2010, with a cure rate of less than 40% [2]. The relatively poor prognosis of ovarian cancer is due to the lack of detection at an early stage and the limited application of effective therapies for the advanced-stage disease [3]. Established clinicopathological prognostic factors of EOC include World Health Organization (WHO) grade, residual tumor after primary surgery, age at diagnosis, performance status, histological characteristics, and tumor rupture during surgery. However these factors inadequately predicted the clinical outcomes of EOC [4]. Recently, several molecular markers that contribute significant roles in the formation and progression of EOC have been identified. According to the NCCN Ovarian Cancer Guideline Version 1.2016, BRCA1 or BRCA2 mutations are high risk carriers of ovarian cancer [5]. A UK based trial compared the multimodality screening of ovarian cancer with ultrasound and CA-125 versus either ultrasound alone and/or no screening and concluded that the former was more effective in the detection of early-stage cancer [6]. Despite this evidence, the molecular mechanisms contributing to its aggressiveness are not fully understood. Therefore, the identification of novel prognostic markers has a substantial clinical impact on the future management of EOC.

P53 is a tumor suppressor protein that is widely studied as a prognostic factor of EOC. In addition, the EGFR and HER-2/neu proteins are considered prognostic factors of EOC. These markers have been used in cancer therapy. HER-2 (p185, HER-2/neu, ErbB-2) is a tyrosine kinase receptor that is located in the cell membrane and is 185 kDa in size. TheHER-2 receptor regulates a multitude of biological processes such as cell proliferation, apoptosis, survival, migration, and differentiation [7].

Slamon investigated the association of HER2 overexpression with the survival of EOC patients [8]. The majority of the subsequent studies further support the hypothesis that HER-2/neu overexpression is an adverse prognostic factor for the survival of patients with EOC [9–14]. However, potential heterogeneity existed among these studies, and hence the prognostic value of the elevated expression of HER-2/neu was not investigated.

Therefore, this systematic review and meta-analysis study was conducted in order to comprehensively combine the available study findings on the effects of HER-2/neu expression on the EOC patient survival.

## RESULTS

### Study selection procedure

**Figure 1:**
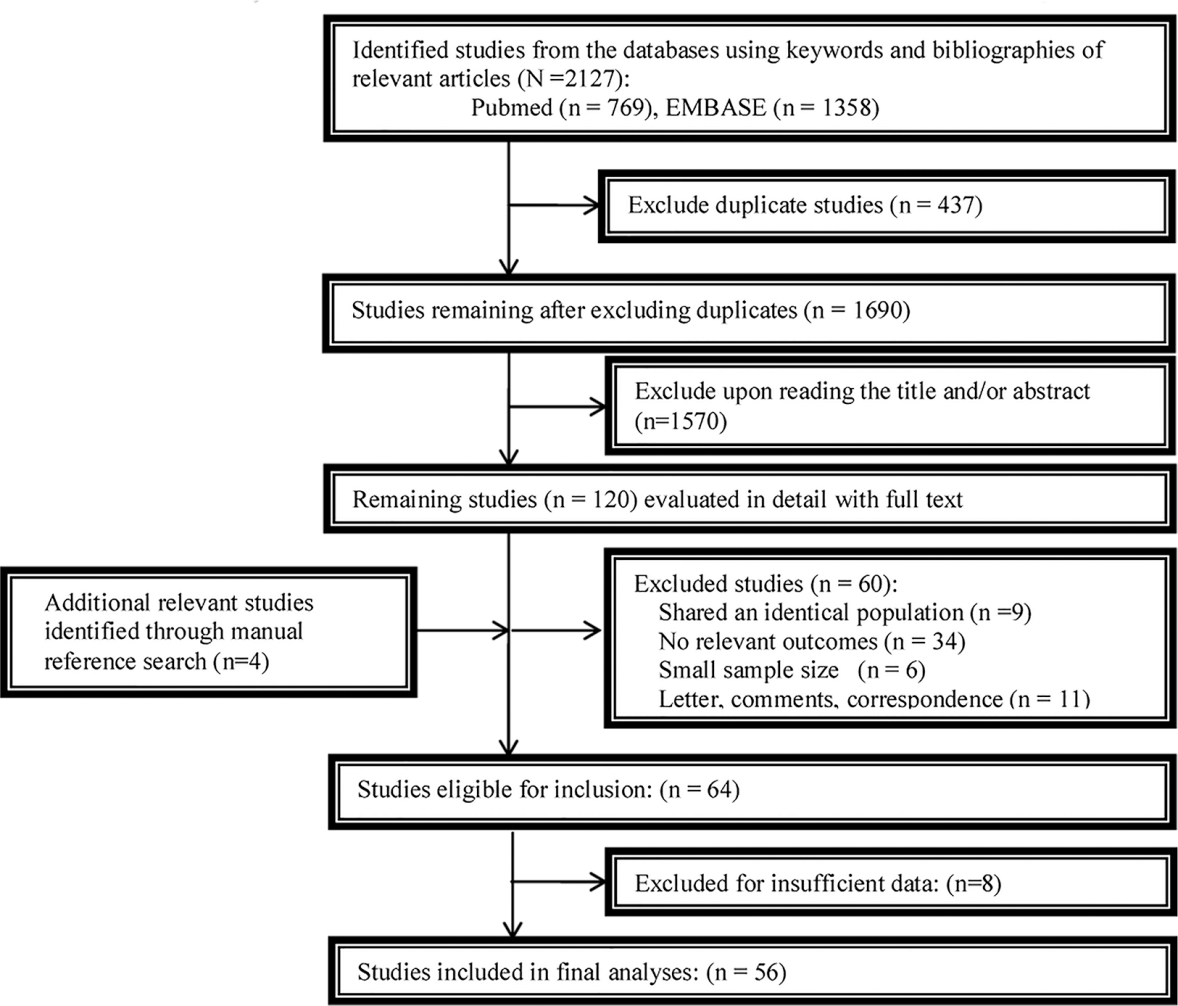
A flowchart of study selection

The study selection procedure was shown in Figure 1. The initial literature search indicated a total of 2,127 studies. Of these, 437 studies were excluded because of overlapping data sets. Subsequently, 1,570 studies were ruled out by reading the title and/or abstract. An additional 4 relevant studies were included from the reference lists. The full text reading of the remaining studies resulted in the exclusion of 60 additional studies (9 studies shared an identical population; 34 studies had no relevant outcomes; 6 studies were with small sample size and 11 studies were letters, comments, or correspondence). Moreover, 8 studies were ruled out due to insufficient data. Finally, 56 studies were included with sufficient data for extraction.

### Study characteristics

The study characteristics are highlighted in Table 1. Finally, a total of 56 studies were included: 8 studies from North America, 7 from Asia, and 36 from Europe [8, 9, 11–64]. These studies were published between the years 1989 and 2015. A total of 7,212 patients were included in the meta-analysis study with a median sample size of 40–783. The median follow-up period ranged from 33 to 213 months. The parameters overall survival (OS) and progression-free survival (PFS) were extracted from 49 and 22 studies, respectively.

**Table 1:** Main characteristics of all the studies included in the meta-analysis

Author	Year	Region	Single or multicenter	No. of patients	mean/median age(ys)	WHO stage	expression detection method	outcomes	Follow up period (years or months or day)	Survival analysis	Adjusted variables	Chemotherapy
Slamon et al. [6]	1989	USA	single	120	NR	NR	IHC/South	OS	median 75.4 months	Univariate	_	_
Berchuck et al. [7]	1990	USA	single	73	NR	III-IV	IHC	OS	NR	Univariate	_	_
Rubin et al. [19]	1993	USA	single	105	mean 59(33-81)	III-IV	IHC	OS	median 34 months	multivariate	Stage, grade, residual tumor, histologicaltype, Residual tumor	Platinum-based
Scambia et al. [20]	1993	Italy	single	94	median 57(14-83)	III-IV	IHC	PFS	median 24 months	Univariate	Age, Stage, grade, Ascites, histological type, Surgical debulking, Response to chemotherapy	Platinum-based
Singleton et al. [21]	1994	USA	single	56	NR	I-IV	IHC	OS	NR	Univariate	stage, histopathologic subtype, grade	Platinum-based
Rubin et al. [22]	1994	USA	single	40	mean 53(26-77)	I-II	IHC	PFS OS	mean 32 months	Univariate	Stage, histological type, grade	_
van Dam et al. [23]	1994	Norwegian	single	80	NR	I-IV	IHC	OS	NR	multivariate	Age, stage, histopathologicsubtype, grade, residualdisease, c-myc, c-ras, EGFR	Platinum-based
Felip et al. [24]	1995	Spain	single	72	Mean 55 (19-74)	I-IV	IHC	OS	median 50 weeks	multivariate	Age, Stage, histological type, Residual tumor, Chemotherapy	Ca.C
Fajac et al. [25]	1995	France	single	65	mean 52	I-IV	Southern Blot	OS	median 71 months	univariate and multivariate	Ag, Stage, grade, histological type, Residual tumor	Platinum-based
Medl et al. [26]	1995	Austria	Multicenter	196	median 59.6(15-88)	I-IV	PCR	OS	mean 59 months	Univariate	Stage, grade, Residual tumor, Ascites, INT-2, ER	Platinum-based
Kaufmann et al. [27]	1995	Germany	single	77	median 63 (33-83)	I-IV	immunoassay	OS	median 19 months	multivariate	age, stage, residual tumor, Ca125, Chemotherapy	Platinum-based
van der Zee et al. [28]	1995	Netherlands.	single	89	mean 50	I-IV	IHC	PFS	NR	multivariate	Age, Stage, grade, Ascites, histological type, P53, Chemotherapy	Platinum-based
Tanner et al. [29]	1996	Germany	single	79	NR	I-IV	S1 Nuclease Assay	OS	NR	multivariate	FIGO stage, histopathologic subtype, grade	P.C/Ca.C
Beckmann et al. [30]	1996	Germany.	single	79	mean52 (34-80)	I-IV	PCR	PFS OS	median 42 months	multivariate	age, stage of disease, grade, c-myc	Ca.C
Meden et al. [9]	1998	Germany	single	208	mean 60	I-IV	IHC	OS	median 24 months	Univariate	Age, Stage, grade, histological type, chemotherapy	P.C/Ca.C
Hengstler et al. [3]	1999	Germany	single	77	NR	I-IV	S1 Nuclease Assay	OS	NR	multivariate	Age, FIGO stage, histopathologic subtype, residual disease, chemotherapy, grade,	Platinum-based
Wang ZR et al. [32]	1999	USA	single	40	median 61 (35-83)	II–IV	FISH	OS	maximum 56 months.	multivariate	Age, Stage, grade, histological type, c-myc	_
Davidson et al. [10]	2000	Israel	single	45	median 56 (30–84)	III–IV	IHC	OS	Mean 70 months	multivariate	Age, histological type, EGFR, E-cadherin, Y-Catenin	_
Seki et al. [33]	2000	Japan	single	48	NR	I-IV	PCR	OS	maximum 87months.	NR	Age, Stage, grade, histological type, tumor size, Ascites, CA-125, residual disease	_
Frutuoso et al. [34]	2001	Portuguesa	single	81	mean 55.4 ± 15	I-III	IHC	OS	NR	Univariate	Age, Stage, grade, Residual tumor, histological type, P53	_
Skirnisdottir et al. [35]	2001	Sweden	single	106	mean 60	IA-IIC	IHC	OS	median87	Univariate	mean age, FIGO stage and histopathologic subtype, grade, EGFR	Platinum-based
Li et al. [36]	2002	china	single	84	median 49	I-IV	IHC	OS	median 32.8 months	Univariate	age, FIGO stage and histopathologic subtype, grade, P53,	_
Hogdall et al. [37]	2003	Danish	Multicenter	181	median 60	I-IV	IHC	0s	NR	multivariate	_	_
Tomic et al. [38]	2003	Croatia	single	80	median 59 (34-79)	I-IV	IHC	OS	median 21month	multivariate	age, stage, grade, p53, nm23, Vascular invasion	_
Camilleri-Broet et al. [39]	2004	Europe	Multicenter	117	median 59 (18-70)	III-IV	IHC	OS PFS	median of 68 months	multivariate	age, stage, Ascites, grade, Tumor type, P53, BCL-2, receptors	P.E.C
Nielsen et al. [40]	2004	Denmark	Multicenter	783	median 58(13-91)	I-IV	IHC	OS	median 17.8 years	multivariate	age, stage, grade, histological type, P53, EGFR	P.A.C/Ca.C
Riener et al. [41]	2004	Germany	Multicenter	361	median 57.6	IIB–IV	IHC	OS, PFS	median 49.1 months	Univariate	Stage, grade, histological type, residual tumor, lymph node metastasis	Platinum-based
Tanabe et al. [42]	2004	Japan	Multicenter	90	NR	I-IV	IHC	OS	NR	Univariate	Stage, histological type, lymph node metasta-sis	_
Elie et al. [11]	2004	France	multicentre	93	Median 58	III-IV	IHC	PFS OS	median 69 months	multivariate	Age, Stage, grade, Ascites, histological type, Residual tumour	Platinum-based
Chan et al. [43]	2004	USA	multicentre	46	mean 41 years	III–IV	IHC	OS	median 37 months	multivariate	Age, Stage, grade, histological type	Platinum-based
Lee et al. [44]	2005	British	single	103	mean 58(35-82)	III-IV	IHC/FISH	PFS	NR	Univariate	_	P.T
Verri et al. [45]	2005	Italy	single	194	Median 57 (25–90)	I-IV	IHC	OS PFS	median 45 months	multivariate	Age, stage, grade, histologicaltype, residual tumor	Platinum-based
Wang et al. [46]	2005	Norway	single	118	median 60(38-81)	II–IV	IHC	PFS	Median 72 months	multivariate	Age, Stage, grade, histological type, residual disease, ER	Platinum-based
Mayr et al. [12]	2006	Germany	single	163	NR	I-IV	IHC/FISH	OS	NR	Univariate	Age, Stage, histological type	_
Surowiak et al. [47]	2006	Poland	single	43	mean 51.0	I-III	IHC	PFS OS	Median 24.6 months	multivariate	Age, Stage, grade, histological type, Clinical response, CA-15, type of chemotherapy	P.C/A.C.C
Castellvi et al. [48]	2006	Spain	single	75	mean 55(20-87)	I-IV	IHC	OS	Median 31months	multivariate	Age, stage, grade, histologicaltype, residual tumor	_
Brozek et al. [49]	2006	Poland	single	53	NR	I-IV	FISH:	OS	NR	Univariate	Age, Stage, grade, histological type, CA-125, chemotherapy	Platinum-based
Steffensen et al. [50]	2007	Danish	single	160	median 54.5 (29–70)	IIB–IV	IHC/FISH	OS	》10 years	multivariate	Age, stage, grade, histological type, residual tumor, COX2 expression	_
Sueblinvong et al. [51]	2007	Thailand	Multicenter	74	mean 46.31(24–67)	I-II	IHC/FISH	OS, PFS	median 46 months	multivariate	Age, Stage, grade, Ascites, histological type, capsular rupture, capsular adherence	_
Tuefferd et al. [52]	2007	France	Multicenter	320	median 58 (25–77)	I-IV	IHC/FISH	OS PFS	NR	multivariate	Age, Stage, grade, Residual tumor, Ascites, histological type, and performance status	_
Sasaki et al. [53]	2007	Japan	single	141	median 53(23-81)	I-IV	IHC	OS	NR	multivariate	Age, Stage, grade, histological type, residual disease, Ascites, type of chemotherapy	CAP
Malamou-Mitsi et al. [54]	2007	Greece	multicentre	95	mean 63	IIc-IV	IHC	OS/PFS	Median 66 months	multivariate	Age, Stage, grade, histological type, residual disease, P53, Bcl-2, type of chemotherapy	P.C
Coronado Martin et al. [55]	2007	Spain	single	124	median 59.2	I-IV	IHC	PFS OS	Median 62.3 months	multivariate	Age, Stage, grade, histological type, Cirugía óptima, P53	_
Pils et al. [56]	2007	Austria	single	128	mean58.6 (27.6–87.2 years).	I-IV	IHC	OS	Median 43.7 months	multivariate	Age, Stage, grade, histological	P.C
Tomsova et al. [57]	2008	Europe	single	116	median 53 ( 27–82)	I-IV	IHC	OS	Median 39 months	multivariate	, Stage, grade, histological type, type of chemotherapy	Platinum-based
de Graeff et al. [58]	2008	Netherlands	single	232	median 57.8 (22–90)	I-IV	immunostain	OS PFS	NR	Univariate	Age, Stage, grade, Suboptimal debulking, EGFR	Platinum-based
Garcia-Velasco et al. [59]	2008	Spain	single	72	median 57 (28–82)	NA	IHC	OS PFS	median 33 months	Univariate	Age, Stage, grade, Residual disease, ER, PR, P53,	P.C/P.C.C/Ca.C
Pfisterer et al. [60]	2009	Germany	multicentre	359	mean 65	IIB-IV	IHC	PFS OS	median 57.5 months	multivariate	Age, Stage, grading, histological type, residual disease, tchemotherapy	Platinum-based
Farley et al. [61]	2009	USA	multicentre	133	median 59.5 (21.7–78.6)	III–IV	ERBB2/CEP17	PFS OS	NR	multivariate	Age, Stage, grade, histological type, residual disease, Ascites, type of chemotherapy	Platinum-based
Ferrero et al. [62]	2011	Italy	single	113	median 62(25-80)	IIb–IV	IHC	OS	NR	Univariate	Age, Stage, grade, histological type, postoperative residual tumor, type of chemotherapy	Platinum-based
Anglesio et al. [63]	2013	Alberta/AOCS/Mayo/Toronto	multicenter	189	mean 57.8 (20–97)	I-IV	IHC/FISH/CIS	OS PFS	median 4.4 Years	multivariate	age, stage, and debulking statu, KRAS mutation status	_
Chay et al. [64]	2013	Singapore	multicenter	133	median 48.3 (15.8–89.0)	I-IV	DISH, IHC	OS PFS	NR	Stage-Adjusted Analysis	Age, Stage, Ethnic group, differentiation, Lymphovascular invasion	_
Demir et al. [65]	2014	Turkey	single	82	median 54 (24–80)	I-IV	IHC	OS	NR	multivariate	Age, Stage, Residual disease, Distant Metastasis	_
de Toledo et al. [66]	2014	Brazil	single	152	mean 55.2	I-IV	IHC	OS, PFS	mean 43.6 months	multivariate	Age, Menopause, BMI, Histology, Grade, Stage, Residual disease, ER, PR, AR, TNEOC expression	Platinum-based
Farkkila et al. [67]	2014	Finland	single	80	median 52 (19–87)	I-III	IHC	PFS	mean 16.8 years	multivariate	stage, GATA4 expression, nuclear atypia	Platinum-based
Cai et al. [68]	2015	China	single	95	NR	I-IV	IHC	OS	NR	multivariate	grade, histology, stage, FASN expression	_

A.C.C, adriamycin+cyclophosphamide+Carboplatin; Ca.C, Carboplatin+cyclophosphamide; COX2,cytochrome c oxidase subunit II; ER,estrogen receptor; EGFR, epidermal growth factor receptor; FIGO, International Federation of Gynecology and Obstetrics; FASN,fatty acid synthase; FISH, fluorescence *in situ* hybridization; GATA4,GATA binding protein 4; IHC, Immunohistochemistry; N, no; NR, not reported; OS, overall survival; PFS, progression-free survival; P.A.C, Cisplatin+adriamycin+cyclophosphamide; P.C, Cisplatin+cyclophosphamide; P.T, Paclitaxel and cisplatin; P53,nuclear protein 53; PR, Progesterone receptor; TNEOC, triple-negative epithelial ovarian cancer; RT-PCR reverse transcription–polymerase chain reaction; Y, yes.

### HER-2/neu expression and OS

**Figure 2:**
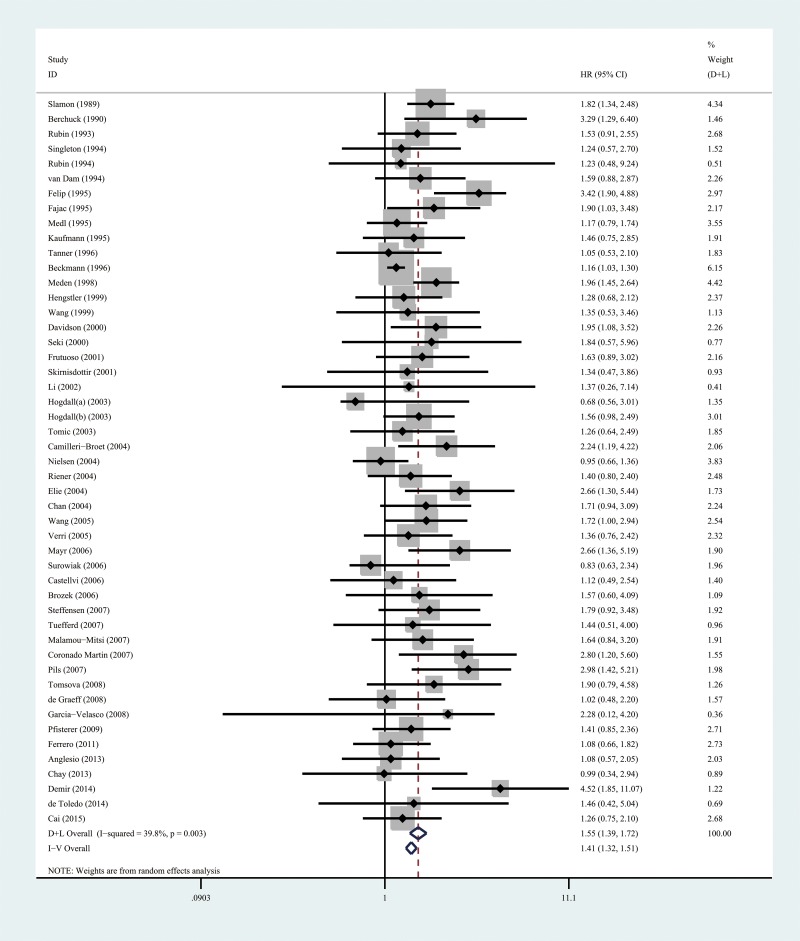
A forest plot of HR and 95% CI of the association between HER-2/neu expression and OS in patients with EOC

Combined analysis showed that the comparison of the patients without HER-2/neu expression with patients possessing HER-2/neu expression always indicated a significant OS disadvantage (HR = 1.41; 95%CI, 1.31 to 1.51; P < 0.001, Table 2, Figure 2). A total of 51 cases were used for this analysis Subgroup analyses were carried out on the basis of the study origin, sample size, follow-up period, patient’s age, detection assay, survival analysis, WHO grade, and chemotherapy regimen in order to overcome the heterogeneity between the studies (*I*^2^ = 39.8, *P* =0.03). Funnel plots indicated significant publication and/or selection biases (*P* = 0.018 for OS; Figure 4A) as demonstrated by substantial asymmetry for HER-2/neu expression.

**Table 2:** Subgroup analyses and meta-regression of the relationships between HER-2 and overall survival or progression-free-survival

Comparison	Overall survival			Progression-free survival		
variables	Number of studies,			Number of studies,		
	Heterogeneity	HR 95%CI, P value	Meta-regression	Heterogeneity	HR 95%CI, P value P value	Meta-regression,
	(I^2^ statistics; %)		P value	(I^2^ statistics; %)		P value
Total	49(39.8)	1.41(1.31 to 1.51),<0.001	NA	22 (32.9)	1.38 (1.23 to 1.56), <0.001	NA
Origin country						
North America	7 (0)	1.74 (1.41 to 2.17), <0.001	0.493	3 (17.6)	1.04 (0.55 to 1.95), 0.525	0.623
Asian	5 (0)	1.47 (1.05 to 2.10), <0.001		2 (0)	1.01(0.47 to 2.17), 0.983	
Europe	37 (48.4)	1.53 (1.34 to 1.74), <0.001		17 (37.2)	1.44 (1.21 to 1.71),<0.001	
Sample size						
≥100	24 (28.3)	1.51 (1.32 to 1.74), <0.001	0.666	13 (34.3)	1.39 (1.12 to 1.72), 0.003	0.990
<100	25 (46.3)	1.60 (1.34 to 1.90), <0.001		9 (37.6)	1.39 (1.10 to 1.80), 0.016	
Median/mean age y						
≥55	29 (34.6)	1.55 (1.35 to 1.78), <0.001	0.823	16 (42.4)	1.46 (1.19 to 1.79),<0.001	0.319
<55	10 (43.6)	1.49 (1.13 to 1.97), 0.005		6 (0)	1.39 (1.18 to 1.63),0.023	
NR	10 (2.7)	1.64 (1.36 to 2.00), <0.001		NR	NR	
Follow up period y						
>5	13 (56.6)	1.64 (1.33 to 2.02), <0.001	0.656	6 (27.5)	1.78 (1.41 to 2.25), <0.001	0.021
<5	22 (28.9)	1.52 (1.30 to 1.79), <0.001		10 (0)		
NR	14 (26.3)	1.51 (1.22 to 1.88), <0.001		6 (40.5)	1.20. (0.98 to 1.47), 0.073	
					1.10 (0.77 to 1.58), 0.595	
detection assay						
IHC	38 (35.0)	1.60 (1.41 to 1.83),<0.001	0.290	20 (30.8)	1.43 (1.19 to 1.71), <0.001	0.411
Others	11 (12.7)	1.33 (1.16 to 1.53),<0.001		2 (71.8)	1.08 (0.56 to 2.10), 0.819	
Survival analysis						
multivariate	34 (47.4)	1.54(1.35 to 1.76), <0.001	0.871	16 (40.2)	1.43 (1.16 to 1.74),0.001	0.549
others	15 (3.9)	1.58(1.34 to 1.73), <0.001		6 (0)	1.23(0.98 to 1.55),0.077	
WHO grade						
II-IV	15 (21.2)	1.79(1.51 to 2.12), <0.001	0.057	9 (52.7)	1.49(1.16 to 1.90), 0.002	0.414
I-IV	34 (34.3)	1.43(1.26 to 1.63), <0.001		13 (13.9)	1.29 (1.04 to 1.61), 0.020	
Centers involved						
Single	36 (45.5)	1.63 (1.42 to 1.87), <0.001	0.155	13 (1.1)	1.38(1.20 to 1.60), <0.001	0.958
Multiple	13 (18.7)	1.55(1.39 to 1.73), 0.001		9 (58.3)	1.35 (0.93 to 1.95), 0.112	
Chemotherapy						
Yes	33 (46.3)	1.50(1.31 to 1.71), <0.001	0.302	18 (39.9)	1.33(1.12 to 1.59), 0.001	0.117
No	16 (0)	1.72(1.46 to 2.03), <0.001		4 (0)	1.94 (1.22 to 3.07), 0.005	

CI, confidence interval; het, heterogeneity; HR, hazard ratio; NA, not available; NR, not reported.

**Figure 3:**
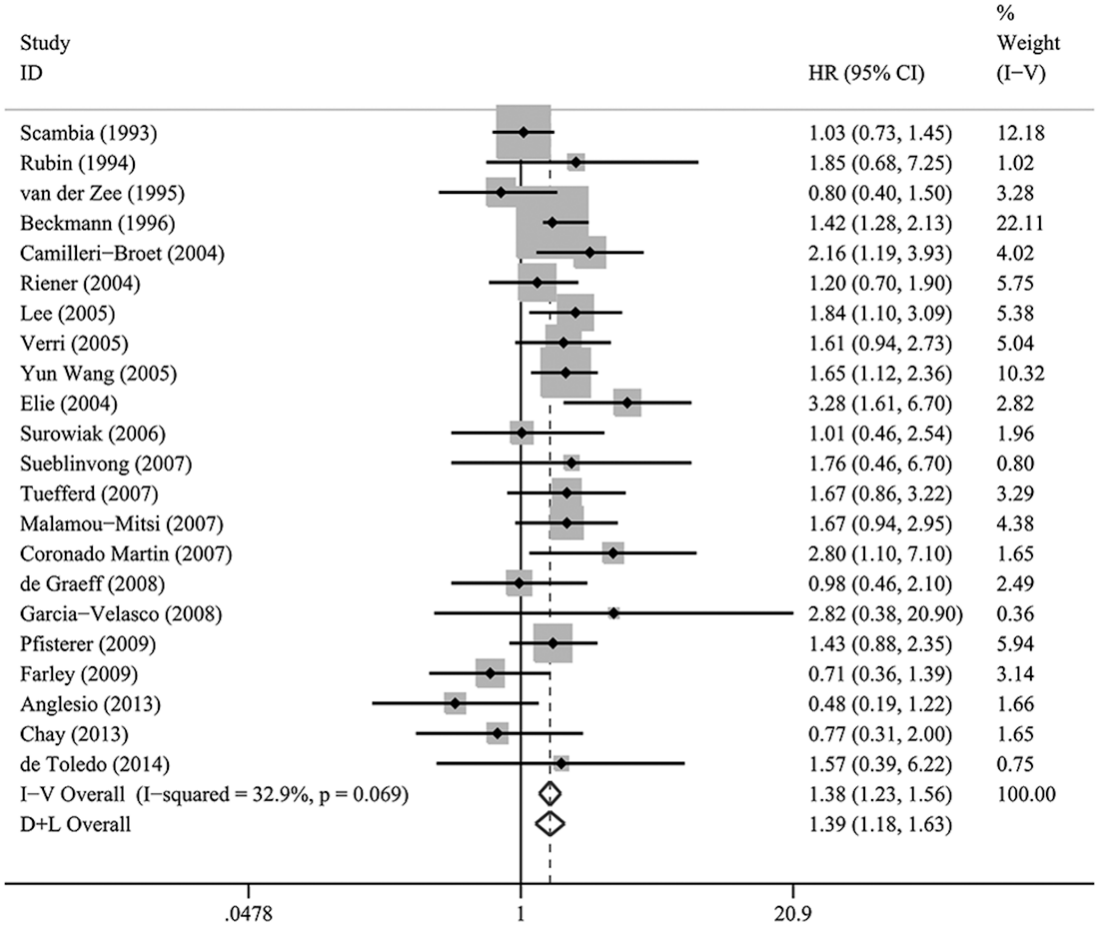
A forest plot of HR and 95% CI of the association between HER-2/neu expression and PFS in patients with EOC

**Figure 4:**
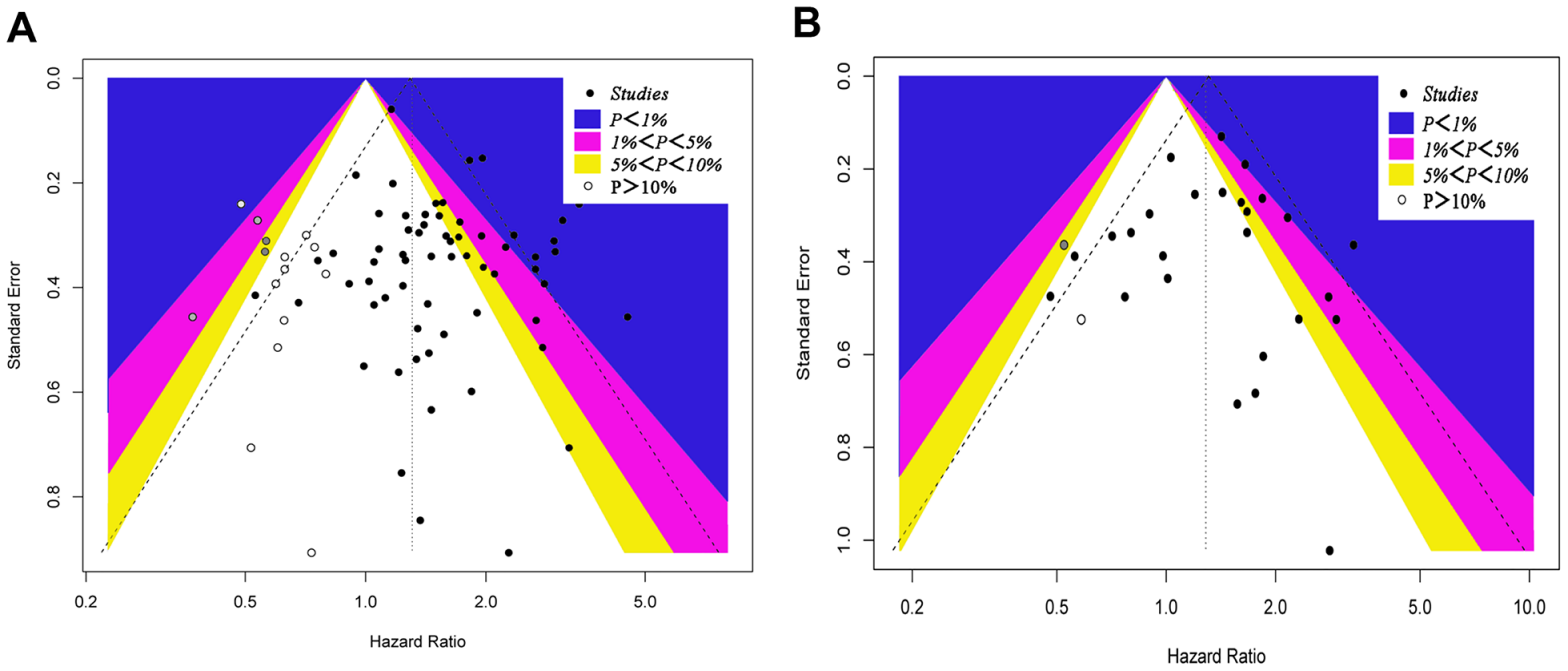
A contour-enhanced funnel plot for meta-analysis **(A)** A contour-enhanced funnel plot for meta-analysis of the association between the HER-2/neu expression and OS in patients with EOC. The left blank area represents the area where 15 studies (white circles) were included when the trim-and-fill method was applied. **(B)** A contour-enhanced funnel plot for meta-analysis of the association between the HER-2/neu expression and PFS in patients with EOC. The left blank area represents the area where two studies (white circles) were included when the trim-and-fill method was applied.

### HER-2/neu expression and PFS

Combined analysis of the included studies showed that upon comparing patients devoid of HER-2/neu expression, the patients possessing HER-2/neu expression demonstrated a significant PFS disadvantage (HR = 1.38; 95% CI, 1.23–1.56; *P* < 0.001, Table 2, Figure 3). The data were derived from 23 studies. Statistically significant heterogeneity was observed between the studies (*I*^2^ = 32.9, *P*=0.069). The investigation of bias demonstrated funnel plot asymmetry for HER-2/neu expression, suggesting the potential of publication and/or selection biases (*P* = 0.037 for PFS; Figure 4B).

### Subgroup analysis and meta-regression

Subgroup analysis was employed in order to explore the heterogeneity causes for OS and PFS. The subgroups that exhibited similar effect sizes were divided into 9 predefined subgroups according to the variables study origin, sample size (≥100 vs. <100), follow-up period, patient’s age, detection assay (IHC vs. Others). The detection assay was defined as ‘Others’ by experimental techniques namely, FISH ELISA and western blotting. The parameters survival analysis (multivariate vs. others), WHO grade (II- IV vs. I-IV) (I: tissue well-differentiated containing many healthy looking cells; II, tissue moderately differentiated with more cells appear abnormal than healthy; III to IV, tissue poorly differentiated or undifferentiated with more cells appear abnormal and lack normal tissue structures), centers involved (single vs. Multiple) and chemotherapy (yes vs. no) were also included. The investigation of the effects caused to the survival of EOC patients by the various study characteristics was conducted by a meta-regression analysis in the subgroups based on HR estimates. For HRs of OS, no statistical significance was noted with regard to the differences in the treatment effects for the subgroups. The P values of study origin, sample size (≥100 vs. <100), follow-up period, patient age, detection assay (IHC vs. Others), survival analysis (multivariate vs. others), WHO grade (II- IV vs. I-IV), Centers involved (single vs. Multiple) and chemotherapy (yes vs. no) were 0.493, 0.666, 0.656, 0.823, 0.290, 0.871, 0.057, 0.155 and 0.302, respectively.

For HRs of PFS, no statistical significance was noted with regard to the differences in the treatment effects for the various subgroups. The P values for study origin, sample size, patient age, detection assay, survival analysis, WHO grade, Centers involved and chemotherapy were 0.623, 0.990, 0.319, 0.411, 0.549, 0.414, 0.958 and 0.117, respectively. However, follow-up period with a P value of 0.021 was identified as variance resource for PFS (Table 2).

### Sensitivity analysis

The heterogeneity that was noted among the included studies with regard to OS was significantly different (I^2^ =49 %). As shown in Figure 2, the studies conducted by Beckmann [24] and Felip et al [8] indicated results that were completely out of range with other studies and probably resulted in the heterogeneity. The Beckmann et al’ study was published in French language, and the Felip et al’ study was conducted in a small sample size. Following exclusion of these studies, the results suggested that patients possessing HER-2/neu expression demonstrated a significant PFS disadvantage over OS (HR=1.375, 95 % CI 1.281-1.476, P<0.0001) compared with patients without HER-2/neu expression. The remaining studies revealed no significant heterogeneity for the variable OS (I^2^= 25.1%).

### Publication bias

Publication bias was evident for OS as demonstrated by funnel plot asymmetry (Figure 4A). A total of 15 missing studies are represented by hollow circles. This finding indicated publication bias, as demonstrated by the Begg’s rank correlation test (*P* = 0.018). The adjusted summary HRs of random-effects was 1.31 (95% CI, 1.17-1.46) and was derived by the trim-and-fill method (TFM) and 1.59 (95% CI, 1.40-1.80). The Copas model confirmed that the analysis was in agreement with the primary analysis of the present study.

As regards PFS, the asymmetric plots were further observed (Figure 4B). TFM did not cause significant alteration to the data when 2 missing studies were included, while the adjusted random-effects summary HR was 1.29 (95% CI, 1.08-1.53). This value was approximately the same with that noted for the summary HR(1.37) (95% CI, 1.10-1.71) that was obtained using the Copas model (Supplementary Table 1).

## DISCUSSION

The present study presented evidence regarding the application of HER-2/neu as a prognostic indicator in EOC subjects. The conclusions were based on pooled data. The data indicated that HER-2/neu expression in EOC subjects had a significant disadvantage on OS and PFS. Concomitantly, the summary of HRs across studies calculated for each subgroup altered the OS results substantially, although the PFS in the subgroups of “North America, Asian” of “country of origin,” “Others” of “survival analysis,” and “Multiple” of “Centers involved” revealed no influence on the results. Finally, the results suggested that the methodology used to assess HER-2/neu expression is considered highly significant. The majority of the meta-analysis studies used IHC staining for the detection of HER-2/neu expression. IHC is a reliable diagnostic technique due to its high sensitivity and specificity, its simplicity and cost-effectiveness. However, the results obtained by IHC are highly dependent on the storage time and fixation method of the paraffin-embedded tissues, the selection of the primary antibody and the IHC staining protocol [65]. Therefore, a subgroup analysis was constructed for the parameter detection assay (IHC vs. Others) and the results demonstrated that different assay methodologies did not change the overall survival (P=0.290).

The present systematic review and meta-analysis have shown that HER-2/neu expression was associated with the survival of EOC subjects. HER-2/neu expression could be used as a prognostic indicator in the majority of the subgroups examined. This finding was based on the parameters sample size, chemotherapy and WHO grade (Table 2). The associations with OS and PFS were similar following adjustment by TFM and/or the Copas model.

In the present study, the observed heterogeneity may be attributed to the positive expression cut off values that ranged from 5% to 65% and the different IHC protocols used. Therefore, the variables IHC staining and scoring protocols have to be taken into consideration during the meta-analysis of the selected studies, due to bias originating from the expression studies of HER-2/neu. The expression of this protein varied among the 45 studies of the present analysis due to different test methods used. These included the type of the primary antibodies and the cut off values used. Thus, it is necessary to establish a uniform methodology for the evaluation of biomarkers.

The studies included and the subgroup analysis indicated the absence of heterogeneity. An additional potential source of bias is attributed to the HR method and the extrapolation of 95% CI. The failure to repeat the latter statistical results may result in additional estimation in the meta analysis by the data presented in each article. In case of the unavailability of the data, the analysis was conducted by data extrapolation from survival curves. Assumptions regarding the censoring process were carried out. The study included multivariate and univariate survival data analysis. These results were further confirmed by an adequately designed prospective study. Moreover, the prognostic merit of the HER-2/neu overexpression status required determination by appropriate multivariate analysis. Several studies contributed to the observed heterogeneity in the meta-analysis of OS and PFS. TFM, the Copas model and the subgroup analyses that were conducted using certain clinical variables, yielded consistent data compared with the primary analyses, indicating the robustness of the data and the lack of publication bias. However, the interpretation of the results should be carried out with caution since publication bias is ubiquitous [66], and cannot be accurately determined by the use of statistical tests.

A total of 3 reviews have previously described the use of HER-2/neu expression as an indicator of the outcomes in EOC [42, 54, 62]. The initial meta-analysis study by P de Graeff et al. demonstrated that the elevated level of HER-2/neu expression was associated with worse overall survival (HR = 1.41; 95%CI, 1.31 to 1.51), although considerable publication bias was present with regard to HER-2/neu expression [40]. The study conducted by Zhao et al. was consistent with the study by P de Graeff, which indicated that HER-2/neu expression was significantly associated with worse PFS and shorter OS. However, publication bias of the studies that were included in the analysis was evaluated by funnel plots and Egger’s test and no significant publication bias was noted. Wang et al. investigated the data from 20 eligible studies in order to evaluate the association of HER-2/neu overexpression and EOC subject survival. The analysis comprised 3,055 patients. Combined HRs suggested that HER-2/neu expression was not significantly associated with survival (HR = 1.05; 95% CI, 0.92–1.19). The Begg’s funnel plot and the Egger’s test were conducted in order to assess the publication bias in the studies.

The present meta-analysis study provides robust statistical evidence regarding the prognosis of EOC subjects determined by HER-2/neu overexpression. Although two previous studies [54, 62] showed a prognostic association of HER-2/neu expression in patients with EOC, the statistical power was low and it was restricted to small sample sizes. The contradiction between the aforementioned studies is attributed to a variety of factors. Firstly, the significant variation in the study design was noted that was notably attributed to the patient recruitment (single center vs. multi center). In addition, certain epidemiological studies were included that used population-based EOC subjects. Secondly, a considerable variation was noted in the sample sizes among different studies. The small sample size renders the studies susceptible to publication bias. The funnel plots (Figure 4) demonstrated non-symmetrical distribution for low statistical power studies. Thirdly, certain differences in the detection assays among studies were noted. IHC was the main detection method. Moreover, the disease characteristics namely, disease stage and tumor location varied among different studies that resulted in heterogeneity with regard to the study findings. In the present study, subgroup analyses were conducted based on certain study characteristics that were found in the prognostic association of tumor HER-2/neu expression. Finally, TFM and the Copas models were applied for adjustment and no significant publication bias was noted, as demonstrated by the consistency of the results.

The present meta-analysis may have several limitations that need to be addressed. Firstly, the possibility of missing studies that are relevant to the topic cannot be avoided, particularly in studies published in languages other than English. Negative studies may exist that were never published as full-length articles, and the original data of several studies could not be obtained. Secondly, the statistical analysis exhibited low power due to the inclusion of limited number of articles (*n* = 56). The present study was based on retrospective studies rather than prospective studies, which limits the potential to effectively avoid recall and selection biases, although the results were adjusted by 2 models (TFM and Copas model). Thirdly, the risk of bias could not be adequately assessed for each publication due to lack of available data for analysis in the majority of the original reports. Moreover, the authors and/or sponsors of certain studies could be contacted for data retrieval [55]. Several HRs in the included studies were from the rough estimates of Kaplan–Meier survival curves [19–21, 25, 29, 64] and therefore the results may be inaccurate. Fourthly, the accuracy and precision of the pooled estimates could be affected by the different survival analysis method. The multivariate Cox proportional hazards model was mainly used by most studies, while a few studies failed to report the statistical model used, and a limited number of studies applied solely univariate analysis [9, 11, 16–18, 30, 31]. In addition, adjustment variables varied considerably across studies. Moreover, the majority of the HRs from the included studies were from multivariate analyses by adjustment for confounding factors. However, different confounding factors were found in different included studies. Therefore, the merged HRs exhibit a degree of heterogeneity. It is important to note that sensitivity analyses related to a patient treatment regimen and/or detailed subgroup analyses could not be conducted according to the tumor site (left EOC or right EOC) and disease stage due to lack of available data in the studies examined. Certain factors such as the aforementioned clinicopathological parameters have been shown to be associated with both HER-2/neu expression and prognosis in EOC patients. Finally, evident heterogeneity was present for several outcomes that could not be explained substantially by the present subgroups. This limits the understanding of the association in various settings and restricts the general ability of the findings.

The present study exhibits significant advantages. A coherent, extensive, and reproducible search of the relevant studies was conducted using several online databases in the absence of limitations such as publication status that resulted in the selection of appropriate studies for this meta-analysis. Furthermore, we included a large sample size of 7,212 patients that enabled the quantitative assessment of the association of HER-2/neu expression with EOC prognosis. Moreover, the subgroup analyses were conducted according to key study characteristics namely, the country of origin, the disease stage, the detection assay, and the follow-up period. Consistent findings were obtained irrespective of the majority of the study characteristics. In addition, although subjectivity was noted in the assessment, the quality scale of the existing prognostic studies provided the rating of the scientific evidence used in the analysis. Finally, the majority of the studies revealed null association with HER-2/neu status, while others provided either negative or positive association, with HER-2/neu status indicating the uncertainty of the survival outcomes of the latter status in EOC patients. The current study minimized the selection bias by the strict pre-specification screening process that was based on the study eligibility criteria. Additionally, multiple modalities were used to evaluate the extent of publication bias.

Taken collectively, the results of the present systematic review and meta-analysis provide strong evidence of the prognostic value of HER-2/neu expression for EOC patients. The study further indicated that HER-2/neu is associated with lower OS and PFS. Therefore, it can be deduced that HER-2/neu targeted therapy is a promising and revolutionary strategy for cancer patients.

## MATERIALS AND METHODS

### Search strategy and study selection

A comprehensive search of the medical literature was conducted on studies evaluating the effect of HER-2/neu expression on the survival of patients with EOC. Databases such as PubMed and Embase were searched using the terms “ovaries” or “ovary” or “ovarian”, “cancer*” or “malignan*” or “tumour” or “carcinoma or neoplas*” or “tumor”, “cerbB2” or “Neu” or “HER2” or “human epidermal growth factor receptor 2”, and “prognos*” or “recurren*” or “death” or “predict*” or “survival”. The literature search was executed in March 2016. Detailed search strategies for both databases are shown in Supplementary Appendix 1. Furthermore, references were searched manually to identify relevant studies during the screening process.

All candidate studies were reviewed by two independent reviewers (Wang K and Zheng LZ), and any disagreement was solved by the third investigator. The search was initially narrowed based on the title followed by the abstract, and finally full papers were reviewed if they were categorized as relevant studies. All references from review papers and original reports were examined to further identify any relevant studies. The inclusion criteria were as follows: (i) studies published as original article, regardless of the language; (ii) studies which involves EOC diagnosis by pathological examination; (iii) studies that reported the correlation of HER-2/neu expression with overall survival (OS) or progression-free survival (PFS); (iv) the papers that did not directly provide hazard ratios (HRs)/odds ratios and 95% confidence intervals (CIs) were kept to rebuild them using the *P* values and other data reported; (v) the most recent studies or comprehensive reports where the same group or author reported results were procured from the same EOC patient population in more than one article; (vi) studies that included more than 40 patients.

### Data extraction

The final data were extracted from the included studies independently by two reviewers (Wang K and Zheng LZ). Data extraction of first author’s name, publication year, country of the population studied, number of patients, age at the time of diagnosis (mean, median, range), WHO grade, assay method, treatment regimen, survival data including OS and PFS, time of follow-up (median, mean, minimum, and maximum), survival analysis, and adjustment variables were performed. OS was defined as the time from the medical treatment until death or last follow-up. PFS was calculated as the interval between the date of treatment and the detection of recurrence or death from any cause. Disagreements between the researchers' were resolved by discussing with a third reviewer (Zheng YQ) until a consensus was reached or by contracting experts if necessary. From studies that reported HR in both univariate and multivariate models, we extracted data from the latter because these results were more convincing, as there had been adjustment for potential confounders. The more significant HR value was extracted instead of both HR values.

### Quality assessment of primary studies

Quality assessment of the included primary studies was independently performed by two reviewers (Wang K and Zheng LZ) using the Newcastle–Ottawa Scale (NOS) [13]. Studies with NOS score ≥6 were considered high quality research. Any disagreement was solved by discussion.

### Statistical analysis

The Stata 12.0 statistical software (Stata Corporation, TX, USA) was used to perform meta-analysis. HR and 95% CI were estimated directly from each study or from an estimation of the Kaplan–Meier survival curves using the methods by Parmar et al [67]. A HR value <1 indicated a better prognosis in patients with EOC and HER-2/neu expression, whereas HR value >1 implied a poor prognosis. If several HR estimates were presented in the same study, only the most powerful ones (multivariate analysis was chosen over univariate analysis, and univariate analysis was chosen over unadjusted Kaplan–Meier analysis) were chosen.

This study investigated the between-study heterogeneity using the Cochran’s Q-test and *I*^2^statistics, and a *P* value for heterogeneity by *I*^2^ value ≥50% suggested substantial heterogeneity. The random-effects model, which is generally more conservative was chosen. Also, subgroup analyses were performed to investigate the potential causes of heterogeneity according to study origin, sample size, follow-up period, patient’s age, detection assay, survival analysis, WHO grade and chemotherapy regimen.

The evidence of publication bias was assessed by the visual judgment of the contour-enhanced funnel plot symmetry as well as by Begg’s regression and Egger’s linear regression methods [68, 69]. Duval’s nonparametric trim-and-fill procedure was applied to assess the possible effect of publication bias [70]. Moreover, the Copas model was used to conduct sensitivity analysis by considering both the effect size and sample size [71]. All statistical tests were two sided, and a *P* value of <0.05 was considered to be statistically significant.

## SUPPLEMENTARY MATERIALS APPENDIX AND TABLE



## Abbreviations

(EOC): Epithelial ovarian cancer
(OS): overall survival
(PFS): progression-free survival
(HRs): Hazard ratios
(CI): confidence interval
(WHO): World Health Organization
(HER-2/neu): human epidermal growth factor receptor 2
(FISH): fluorescence *in situ* hybridization
(ELISA): enzyme-linked immuno sorbent assay
