# Supplementary material for: Prognostic value of HER-2/neu expression in epithelial ovarian cancer: a systematic review and meta-analysis
Source: Oncotarget. 2017 Sep 6;8(43):75528–43. doi: 10.18632/oncotarget.20657 (PMC5650443; doi:10.18632/oncotarget.20657)
Supplement: Supplementary file 1 [file oncotarget-08-75528-s001.pdf]

Prognostic value of HER-2/neu expression in epithelial ovarian cancer: a systematic review and meta-analysis

SUPPLEMENTARY MATERIALS

APPENDIX

Search Strategy

Search included: PubMed, Embase, and the Cochrane Library Central Register of Controlled Trials, till March, 2016.

Supplementary Appendix 1: Search strategy for Pubmed (Publication date to 2016/03/17) result 769

1. “Ovarian Neoplasms” [Mesh]
2. ((Ovarian[Title/Abstract]) OR Ovary[Title/Abstract]) OR ovaries[Title/Abstract]
3. (((((cancer*[Title/Abstract]) OR tumor[Title/Abstract]) OR tumour[Title/Abstract]) OR carcinoma[Title/Abstract]) OR neoplas*[Title/Abstract]) OR malignan*[Title/Abstract]
4. 2 AND 3
5. 1 OR 4
6. “Genes, erbB-2”[Mesh]
7. ((((((c-erbB-2[Title/Abstract]) OR c-erbB2[Title/Abstract]) OR Neu[Title/Abstract]) OR HER2[Title/Abstract]) OR HER-2/neu[Title/Abstract]) OR HER-2[Title/Abstract]) OR human epidermal growth factor receptor 2[Title/Abstract]
8. 6 OR 7
9. “Mortality”[Mesh]
10. “Survival”[Mesh]
11. “Prognosis”[Mesh]
12. ((((((prognos*[Title/Abstract]) OR survival[Title/Abstract]) OR recurren*[Title/Abstract]) OR mortality[Title/Abstract]) OR predict*[Title/Abstract]) OR outcome*[Title/Abstract]) OR death[Title/Abstract]
13. 9OR 10 OR 11 OR 12
14. 5 AND8AND 13

[(ovaries or ovary or ovarian) and (cancer* or malignan* or tumour or carcinoma or neoplas* or tumor)]
(cerbB2 or Neu or HER2 or “human epidermal growth factor receptor 2”)
(prognos* or recurren* or death or predict* or survival).

Supplementary Appendix 1: Search strategy forEmbase(Publication date to 2016/03/22) result 1358

15. 'ovary cancer'/exp
16. ((ovaries or ovary or ovarian) and (cancer* or malignan* or tumour or carcinoma or neoplas* or tumor)):ab,ti
17. 1OR2
18. 'epidermal growth factor receptor 2'/exp
19. (cerbB2 OR Neu OR HER2 OR ‘human epidermal growth factor receptor 2’):ab,ti
20. 4 OR 5
21. 'mortality'/exp
22. 'survival'/exp
23. 'prognosis'/exp
24. (prognos* OR recurren* OR death OR predict* OR survival):ab,ti
25. 7 OR 8 OR 9 OR 10
26. 3 AND 6 AND 11

Supplementary Table 1: Meta-analysis of HER-2/neu expression and patient survival outcomes; analyses of the publication bias with different models for the overall survival and progression-free survival

Variable	No of studies	HR(95% CI)	P
Overall survival	51	1.55 (1.40 to 1.72)	<0.001
Progression-free survival	23	1.20 (0.98 to 1.46)	<0.001
**Publication bias**	**Begg's *P* value**	**Egger's *P* value**	**T&F(Fill)**
Overall survival	0.808	0.018	1.31 (1.17–1.46)
Progression-free survival	0.402	0.037	1.29 (1.08–1.53)

Abbreviations: CI, confidence interval; Fill, number of studies added by trim-and-fill method; het, heterogeneity; HR, hazard ratio; T&F, result of trim-and-fill analysis, using assumption of random effects.
